# RELIGIOSITY/SPIRITUALITY PROMOTES POSITIVE WELL-BEING FOR MIDDLE-AGED ADULTS IN THE CONTEXT OF MONTHLY ADVERSITY

**DOI:** 10.1093/geroni/igad104.2730

**Published:** 2023-12-21

**Authors:** Nutifafa Dey, Frank Infurna, Kevin Grimm

**Affiliations:** Arizona State University, Tempe, Arizona, United States; Arizona State University, Tempe, Arizona, United States; Arizona State University, Tempe, Arizona, United States

## Abstract

Religiosity/Spirituality (R/S) are long established in aging research to protect against adversity and promote positive development. However, little about this is known regarding whether and which components of R/S (interpersonal and intrapersonal) in the context of longitudinal research in middle-aged adults. Using data from an intensive longitudinal study of 362 middle-aged adults (ages 50–65) assessed monthly over a period of two years, we applied multilevel models to examine whether R/S at the between- and within-person levels are a protective resource against the detrimental effects of monthly adversity on psychological well-being. Reporting more R/S at monthly assessments (within-person) where adversity was reported was associated with reporting fewer depressive symptoms and negative affect, but was not protective for the outcomes of life satisfaction and positive affect. Considered separately, the two domains of R/S protected against adversity’s impact across each of the outcomes. Reporting more between-person levels of intrapersonal R/S (mapping strongly onto spirituality) did not protect against adversity, but instead exacerbated the detrimental effects of adversity on each outcome. The effects observed here imply that on monthly occasions where individuals report more R/S, this will lead to less of a negative impact of monthly adversity on each outcome. Also, at the between-person level, individuals who, on average, report more involvement in R/S will show less detrimental effects of monthly adversity on each outcome; yet this may not be true for intrapersonal R/S. We further elaborate R/S’s protection and offer some explanations for why between-person changes in intrapersonal R/S exacerbates adversity.

